# Diagnostic and Interventional Radiology for Liver Diseases

**DOI:** 10.1155/2015/147583

**Published:** 2015-03-24

**Authors:** Satoru Murata, Pascal Niggemann, Edward W. Lee, Per Kristian Hol

**Affiliations:** ^1^Department of Radiology/Center for Advanced Medical Technology, Nippon Medical School, 1-1-5 Sendagi, Bunkyou-ku, Tokyo 113-8602, Japan; ^2^Department of Radiology, Diakoniekrankenhaus Mannheim, Speyererstr. 91, 68163 Mannheim, Germany; ^3^Department of Radiology, UCLA Medical Center, David Geffen School of Medicine at UCLA757, Westwood Plaza, Suite 2125B, Los Angeles, CA 90095, USA; ^4^The Intervention Centre, Oslo University Hospital Rikshospitalet, Pb 4950 Nydalen, 0424 Oslo, Norway

Diseases of the liver are common and often chronic. Moreover, the mortality rate associated with chronic liver diseases remains high, despite the constant development of novel diagnostic and therapeutic modalities; therefore, numerous efforts are being made to improve imaging techniques, especially in this decade. Currently available imaging procedures allow us to ascertain the morphology, circulation, metabolism, parenchymal texture, fibrosis, and/or tumor viability in the liver. New modalities and protocols, such as magnetic resonance (MR) perfusion, MR elastography, and dual-energy computed tomography (CT), enable the potential evaluation of liver function via imaging studies. Thus, the utilization of advanced imaging techniques and contemporary interventional radiology (IR) devices has realized novel multimodality treatments for liver diseases, resulting in promising outcomes in many patients who cannot be surgically treated.

This special issue of BioMed Research International reviews recent diagnostic and interventional radiological aspects of various liver diseases such as liver cancer, fibrosis, chronic hepatitis, liver steatosis, and portal hypertension. In particular, new advances in imaging devices and protocols to evaluate fibrosis or hepatitis are described, as well as functional magnetic resonance imaging (MRI) techniques for the liver. On the other hand, various interventional radiological techniques to provide more efficient therapy to patients with advanced liver cancer are also introduced.

MRI is often performed to assess the liver in patients with chronic liver diseases. In this special issue, B. S. Kim et al. present a well-written review on a range of most utilized liver MR sequences to image patients with poor breath-hold capabilities. Recent updates on robust liver imaging as well as the advantages and disadvantages of these new methods are discussed in detail.

Liver fibrosis is a life-threatening condition with high morbidity and mortality owing to its diverse causes. Liver biopsy is the gold-standard method for diagnosing and staging liver fibrosis in chronic liver diseases, but it has several limitations, including sample variability and its invasive nature with potential complications. To resolve these problems, different noninvasive imaging-based methods have been developed for the accurate diagnosis of liver fibrosis. However, these techniques can only evaluate morphological or perfusion-related alterations of the liver, and thus, they are useful for the diagnosis of only late-stage liver fibrosis, which is characterized by “irreversible” anatomic and hemodynamic changes. Therefore, the early identification of hepatic fibrosis is of clinical significance to timely initiate therapy and to effectively achieve disease regression. In this special issue, S. Palmucci et al. and Z. Li et al. review liver fibrosis evaluated by diffusion-weighted MRI and molecular MRI techniques, respectively, to offer valuable perspectives on the development and limitations of diagnosing early-stage liver fibrosis.

The two MRI-related original research reports in this issue are authored by J. M. Alústiza et al. and F. Paparo et al. who describe MRI liver iron quantification by using the liver-to-muscle ratio and report the reproducibility of such a method on different MRI machines. Their results confirm its practicality and suggest the possibly wider acceptance of this elegant noninvasive technique. F. Paparo et al. report that MRI proton density fat fraction is a useful technique for the noninvasive assessment of liver steatosis in patients with chronic viral C hepatitis.

Diagnostic imaging is increasingly being performed to enable the treatment of liver diseases, and this trend is expected to persist. Hepatectomy is considered the first choice of treatment for early hepatocellular carcinoma (HCC) and resectable cholangiocellular carcinoma. Although extended resection is sometimes required for a cure, a sufficient volume of the remnant liver should be preserved, unless hepatic failure ensues after surgery. Portal vein embolization (PVE) is an established and effective method to increase the volume of the future liver remnant and allows more extensive resections. In this issue, A. Akiba et al. describe the usefulness of gadolinium-ethoxybenzyl-diethylenetriamine pentaacetic acid (Gd-EOB-DTPA) MRI for the prediction of liver volume change after PVE. They evaluated signal intensity (SI) contrast between nonembolized and embolized areas after PVE as well as the change in SI contrast before and after PVE (SI ratio) to conclude that either parameter had a negative correlation with the percentage of the future liver remnant. Such a result indicated that EOB-MRI might be useful for the prediction of hepatic hypertrophy after PVE.

Many IR techniques have been introduced for the treatment of liver cancers in the last few decades. Transarterial chemoembolization (TACE) is a major IR method to treat unresectable HCC. However, there is an ongoing controversy regarding which chemoembolization materials should be used to achieve good tumor control and reduce side effects. D. Yasui et al. reported the superior efficacy of TACE with warmed miriplatin compared to nonwarmed miriplatin. Because miriplatin, a recently developed anticancer drug with few toxic side effects on the vessel wall during arterial injection, is highly viscous, it yielded suboptimal tumor response. Thus, the study by D. Yasui et al. is significant in demonstrating how to increase the efficiency of TACE with miriplatin. Maximizing TACE visualization of the hepatic tumor and identification of tumor feeding vessels is very important and may require repeated injection of contrast media, possibly leading to renal failure. J. Paul et al. describe an ultrafast cone-beam CT imaging protocol during image-guided hepatic TACE, which reduces the required volume of contrast media and radiation dose, thus allowing more extensive treatment.

TACE results in both tumor hypoxia and longer activity periods of anticancer drugs trapped in the tumor tissue. However, it also induces a posttreatment surge of angiogenic factors, such as vascular endothelial growth factor (VEGF), as early as a few hours after the procedure. Such a process may contribute to tumor revascularization, thus reducing the efficacy of TACE. Therefore, several researchers have combined sorafenib, an antiangiogenic drug that blocks tumor cell proliferation and angiogenesis by inhibiting the activity of VEGF receptors, with TACE to potentially improve treatment outcomes. However, sorafenib cannot be used in patients with severe thrombocytopenia, one of the complications of hypersplenism, owing to its platelet-decreasing effect. Y. Ooka et al. evaluated the long-term outcome of partial splenic embolization (PSE) with selective TACE in patients with advanced HCC accompanied by severe thrombocytopenia and reported that the procedure allowed these patients to receive additional sorafenib chemotherapy.

Radiofrequency ablation (RFA) is known to be an effective minimally invasive treatment for small HCC. However, its efficacy is equivocal for HCC larger than 3 cm, and RFA-related complications might depend heavily on the lesion location. A. Orlacchio et al. demonstrated that RFA with careful preprocedural planning could be safely performed even for lesions larger than 3 cm located in close proximity to the gallbladder. They report a complete necrosis rate of 87% without major complications in a small patient cohort.

In conclusion, the present special issue summarizes recent advances in both diagnostic radiology and interventional radiology, providing us with valuable perspectives in this ever-progressing field.

*Satoru  Murata* *Pascal  Niggemann* *Edward  W.   Lee* *Per  Kristian  Hol*

